# The Importance of Early Imaging in Acute Pyelonephritis: A Case Report of Emphysematous Pyelonephritis

**DOI:** 10.7759/cureus.37132

**Published:** 2023-04-04

**Authors:** Ali Abdulsahib, Mohammad Abu-Abaa, Ghassan Al-Qaysi, Kateryna Chepenko, Deepika Suleria

**Affiliations:** 1 Internal Medicine, Capital Health Regional Medical Center, Trenton, USA

**Keywords:** types 2 diabetes, severe sepsis, obstructive pyelonephritis, bilateral nephrostomy tube, extended spectrum-beta lactamase (esbl), emphysematous

## Abstract

Emphysematous pyelonephritis (EPN) is a rare life-threatening infection that is usually encountered in diabetic patients. Herein, we are reporting a 41-year-old male patient with a past medical history of stage 3B chronic kidney disease (CKD), neurogenic bladder, and poorly controlled diabetes who presented with left-sided pyelonephritis and septic shock. *E. coli *was detected in urine and blood. Lack of adequate clinical response to appropriate antibiotic coverage prompted computed tomography (CT) scan of the abdomen that revealed EPN. Despite aggressive conservative management along with nephrostomy, the patient had multiple risk factors to fail conservative management and require nephrectomy. This left the patient on life-long dependence on hemodialysis. This case report is not only interesting as EPN is a rare clinical pathology, but it also helps to remind clinicians to remain vigilant on when to consider early imaging in pyelonephritis. In the appropriate clinical scenario of acute pyelonephritis in a diabetic patient with urinary obstruction, it is important to rule out EPN as an early diagnosis and conservative management including relief of urinary obstruction can lead to a better outcome, help preserve renal function, and spare nephrectomy.

## Introduction

Emphysematous pyelonephritis (EPN) is a rare life-threatening necrotizing infection of renal parenchyma and perirenal tissue. It can be easily recognized on imaging due to its hallmark gas formation within the renal tissue [1]. The two main prerequisites for the development of EPN are infection by a gas-forming bacterium such as *E. coli *or *Klebsiella pneumoniae *and the presence of diabetes with/without urinary tract infection [2]. EPN occurs in patients who have had renal transplantation; men are more susceptible to this infection than women [3]. Depending on the patient’s response to conservative treatment, the need for radical nephrectomy can be determined [2]. This condition requires special attention, especially in patients who have a baseline impaired renal function. This infection should be differentiated from emphysematous pyelitis, in which the infection is limited to the renal collecting system and spares the renal parenchyma [1].

## Case presentation

A 41-year-male patient presented to the emergency department (ED) with abdominal pain, nausea, vomiting, and generalized weakness for five days prior to presentation. Past medical history was significant with uncontrolled type 2 diabetes secondary to poor compliance and stage 3B chronic kidney disease. The patient was also recently diagnosed with bilateral hydronephrosis secondary to a neurogenic bladder, which was treated using an indwelling urinary catheter. Vitals recorded in the ED included a blood pressure of 90/50 mmHg, pulse rate of 101 beats per minute, respiratory rate of 14 cycles per minute, SpO2 of 96%, and temperature of 37°C. Physical examination revealed that he was lethargic but fully oriented and cachectic, with a left costovertebral angle and flank tenderness along with suprapubic tenderness. Otherwise, his exam was unremarkable. Basic labs showed a white blood cell (WBC) count of 12,000 cells/ml, elevated bands of 57%, elevated lactic acid of 3.3 mmol/L, acute kidney injury (AKI) with elevated serum creatinine at 10.32 mg/dl from a baseline of 2.2 mg/dl one month prior, hyperglycemia of 669 mg/dl with anion gap metabolic acidosis of 23 mEq/L, and low bicarbonate of 10 mmol/l (see Table 1). The chest x-ray was clear. The patient was given a 3-liter fluid bolus, after which blood and urine cultures were collected and broad-spectrum antibiotics including vancomycin and cefepime were empirically initiated along with vasopressor therapy. Also, diabetic ketoacidosis (DKA) management was initiated including insulin and fluid therapy in the Intensive Care Unit (ICU). Sodium bicarbonate infusion was also initiated.

**Table 1:** Lab Work Trend on Admission, and on Days 2, 3, and 7

Event name	Admission	Day 2	Day 3	Day 7	Normal range
Hematocrit	26%	23.9%	21.1%	31.1%	40-51%
Hemoglobin	8.7	8.3	7.4	10	13.7-17.5 g/dl
WBCs	12.5	17.6	17.3	33	4-11x 10^3
Platelets	103	73	21	296	150-400x10^3
Sodium	127	133	134	135	137-145 mmol/L
Chloride	94	103	101	98	98-107 mmol/L
Bicarbonate	10	12	18	24	22-30 mmol/L
Glucose	669	206	176	192	70-100 mg/dl
BUN	128	118	120	36	9-20 mg/dl
Creatinine	10	8.9	8.7	4.5	0.60-1.25 mg/dl
Potassium	4.1	3.4	3.8	3.3	3.5-5.1 mmol/L
Lactic acid	3.3	1.3			0.7-1.9 mmol/L

**Figure 1:**
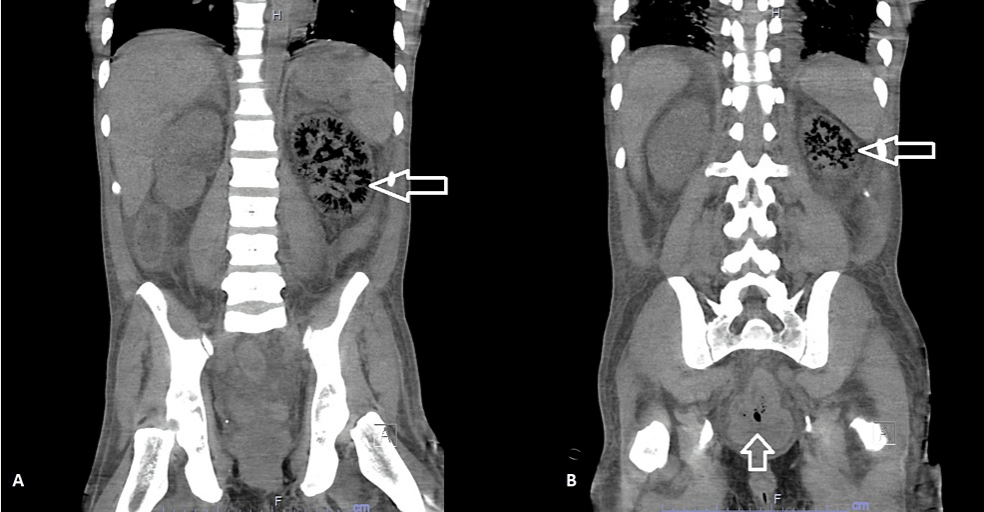
First CT Scan of the Abdomen and Pelvis Showing Extensive Gas Within the Left Renal Collecting System and Parenchyma Along With Intravesical Gas

Several hours later, urine culture revealed *E. coli *with a detectable extended-spectrum beta-lactamase (ESBL) gene. This was resistant to cefepime with a minimum inhibitory concentration (MIC) of more than 32. The same result was seen in blood culture. This prompted the switching of antibiotic coverage to meropenem. However, over the next 24 hours, the vasopressor requirement progressed, which prompted the consideration of computed tomography (CT) scan of the abdomen and pelvis. The scan showed extensive gas within the left renal collecting system and parenchyma along with intravesical gas (Figure 1). The left side nephrostomy tube was placed more than 24 hours after admission. This allowed for partial improvement in renal function and reduced the creatinine down to 8 mg/dl. Although the vasopressor requirement improved, the patient remained oliguric with the progression of thrombocytopenia with a nadir of 22,000 cells/ml and leukocytosis. 

**Figure 2:**
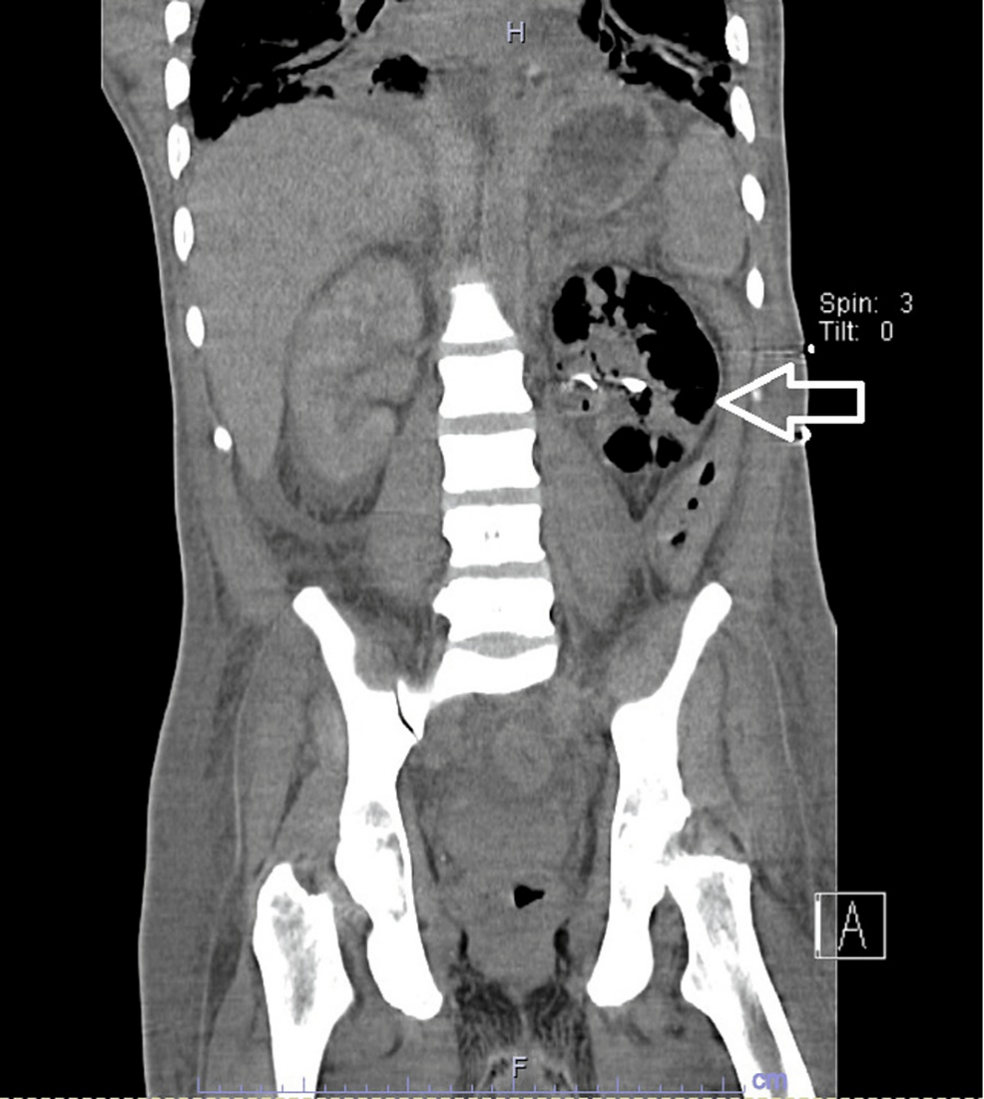
CT Scan of the Abdomen and Pelvis, 48 Hours Post Left Nephrostomy Tube Placement, Showing Progression of EPN in the Left Kidney (Arrow)

**Figure 3:**
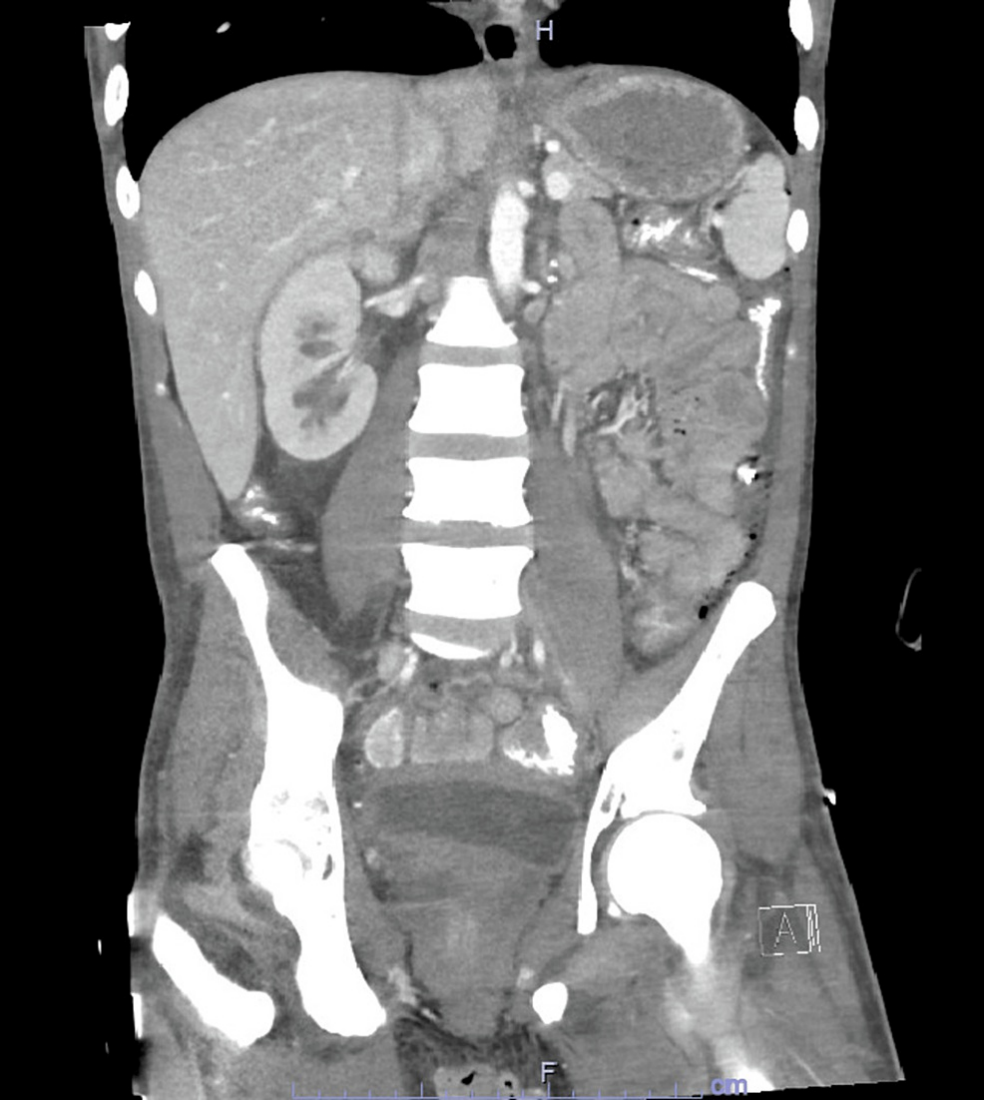
Post Nephrectomy CT Scan Post left-sided nephrectomy CT scan of the abdomen coronal section.

A repeat CT scan of the abdomen and pelvis taken 48 hours later showed a progression of the left EPN to affect the entire renal parenchyma (Figure 2). Persistently elevated creatinine along with refractory metabolic acidosis prompted the initiation of hemodialysis. Open nephrectomy was deferred till an improvement of thrombocytopenia was observed seven days after the initial presentation. The patient remained hemodialysis-dependent. A CT scan taken a few days later showed no further gas evidence (Figure 3). Clinical improvement allowed for a safe discharge. Meropenem was continued for a total of two weeks duration.

## Discussion

Most cases of EPN have been reported in patients who are in the fourth or fifth decades of life [4]. The clinical presentation of EPN is no different from pyelonephritis. Clinical crepitus has been reported only in extremely rare cases, constituting about 8% of the total cases [1]. The left kidney is most commonly affected in 52% of the cases, while right kidney involvement has been reported in 37.7% of the cases. Bilateral involvement has been seen in 10.2% of the cases [5]. An important clinical clue to diagnosing EPN is the lack of clinical response of pyelonephritis to appropriate antibiotics. This should prompt early consideration of a CT scan of the abdomen. CT scan is the test of choice as it allows better determination of the gas amount, renal parenchymal destruction, presence of fluid and air-fluid levels as well as urinary tract obstruction [2]. Ultrasound is less preferred, as it shows renal enlargement with high amplitude echoes.

The main risk factor for EPN is diabetes mellitus, in more than 90% of the cases, and female gender with a female-to-male ratio of 3:1 [1,6,7]. This was revealed in a retrospective study involving 26 patients. Other risk factors include urinary obstruction and immune incompetence [8]. This study showed that the bacterium that is most commonly involved in causing EPN is *E. coli* (in more than 50% of the cases) [1]. Other reported bacteria are *Proteus mirabilis*, *Klebsiella pneumoniae*, *Enterococcus*, and *Pseudomonas aeruginosa* [9]. *Candida albicans* as well as *Acinetobacter* were also reported in some patients [1]. Other risk factors reported were drug abuse, neurogenic bladder, alcoholism, and anatomic abnormalities [4]. EPN usually results from anaerobic metabolism, which is facilitated by high tissue glucose levels combined with impaired blood supply [4]. This results in the fermentation of glucose and lactate-producing gas, mainly carbon dioxide and hydrogen [4].

EPN can be classified into four classes. In class 1, the gas is limited to the pelvicalyceal system; in class 2, the gas extends to the renal parenchyma; in class 3A, the gas extends to the perinephric space; and in class 3B, the gas extends to the pararenal space. Bilateral EPN or EPN affecting a solitary kidney is classified as class 4. The authors who put forth this classification also recommend class-based management [2]. Gas can extend as far as the scrotal sac and spermatic cord [4]. In all classes, a therapeutic approach consisting of appropriate antibiotics, percutaneous drainage, and urinary tract obstruction relief/stenting should be sought [2].

CT scan of the abdomen and pelvis is the cornerstone in the diagnosis and classification of EPN. The need for nephrectomy can be potentially avoided by early recognition and management. Hence, this case report reminds clinicians to remain cognizant of this potentially life-threatening infection [10]. In the present case, a CT scan should have been taken at the time of admission, given the presence of pyelonephritis with all risk factors of EPN including poorly controlled diabetes, urinary obstruction, and infection by *E. coli*.

Conservative medical therapy should be attempted first. However, the point at which nephrectomy should be offered is debatable. Initial recommendations include radical nephrectomy for patients who present with extensive EPN and two or more poor prognostic factors. Poor prognostic factors include septic shock, acute renal impairment, extension of infection to perinephric tissue, severe proteinuria, thrombocytopenia, and altered mental status [2]. However, this has been challenged in more recent studies [1,11]. Our patient presented with three risk factors, namely, acute kidney injury (AKI), thrombocytopenia, and septic shock. Early studies of EPN mentioned a mortality rate of up to 80%, which has recently reduced to 21% [6,12]. It is reported that the mortality rate is higher in nephrectomy as compared to the conservative approach.

## Conclusions

When a patient presents with an appropriate clinical setting and risk factors for EPN, especially diabetes, the lack of clinical response of pyelonephritis to appropriate antibiotics should prompt early consideration of a CT scan of the abdomen to rule out EPN. A conservative approach including antibiotic therapy and percutaneous drainage is recommended initially to preserve renal function. However, failure to improve or worsening of clinical/radiological status should prompt nephrectomy.
